# Normalization in hemophilia: conceptual foundations and clinical implications

**DOI:** 10.1016/j.rpth.2025.103200

**Published:** 2025-09-30

**Authors:** Kazimieras Maneikis, Evelien Krumb, Cedric Hermans

**Affiliations:** 1 Clinic of Hematology and Oncology, Institute of Clinical Medicine, Faculty of Medicine, Vilnius University, Vilnius, Lithuania; 2 Division of Haematology, Haemostasis and Thrombosis Unit, Cliniques Universitaires Saint-Luc, Université Catholique de Louvain (UCLouvain), Brussels, Belgium

**Keywords:** disease burden, hemophilia, hemostasis, normalization, treatment expectations

## Abstract

Hemophilia is an inherited bleeding disorder characterized by a deficiency in clotting factor, leading to impaired thrombin generation, bleeding complications, and long-term morbidity that severely impacts patients’ quality of life. Recent advancements in treatment have significantly empowered the hemophilia community, improving disease control, reducing treatment burden, expanding access to care, and lowering the risks of lifelong complications. These advancements have continually elevated the goals of hemophilia care. The latest innovations have introduced a new ambition: the “normalization” of life for persons with hemophilia. This aspiration is both desirable and promising, with the potential to greatly enhance patients’ quality of life and achieve equitable health outcomes. However, it is crucial for the entire community to first determine and define what constitutes an optimal and realistically achievable level of hemostasis normalization using current and emerging therapies. The significant psychosocial impact of hemophilia underscores the importance of targeting not only hemostasis normalization but also life normalization. This approach prioritizes reducing treatment burden and restoring mental well-being. Novel standardized tools are urgently needed to appropriately complement novel therapies, as traditional bleeding rate-based outcome metrics are becoming inadequate for assessing treatment superiority objectively. The potential risks of striving for normalized hemostasis must also be carefully considered. Moreover, specific needs and challenges associated with normalization must be addressed, particularly for carriers and women and girls with hemophilia.

## Essentials

• Hemophilia is a lifelong bleeding disease significantly affecting hemostasis and patients’ lives.
• Treatment ambitions are persistently increasing with the advent of novel therapies.
• Hemostasis correction warrants careful consideration of its potential pitfalls.
• Normalization of hemostasis alone does not grant full normalization of life.

## Graphical abstract

**Figure:**
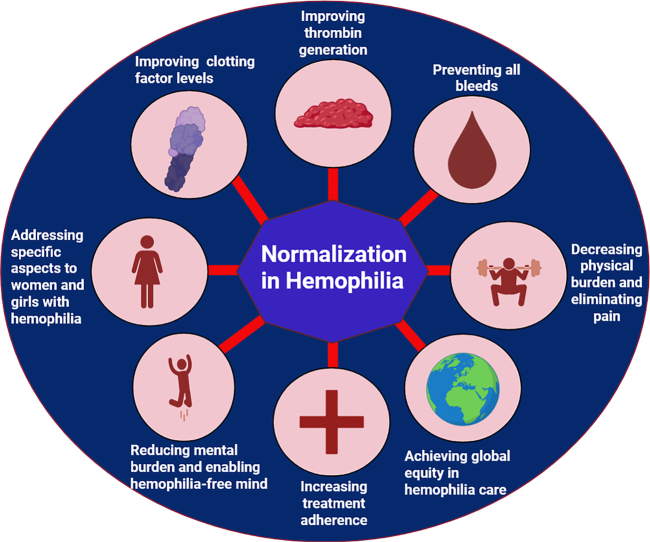

The concept of normalization in hemophilia requires careful consideration of multiple domains, each of which plays a crucial role in determining the most effective treatment strategy for reaching full normalization

## 1 Introduction

Hemophilia has long been a devastating disease, causing significant mortality, morbidity and a major reduction in the quality of life (QoL) [1]. Major therapeutic advancements have enabled better care, granting improved clinical outcomes and empowering many persons with hemophilia to live an active life without being constantly concerned about possible disease complications [2]. Despite substantial progress, treatment remains challenging, and the lives of persons with hemophilia still differ markedly from people without bleeding disorders.

Over the recent decades, treatment advances have shifted the goals of hemophilia care from providing treatment for complete prophylaxis for all patients to achieving zero bleeds for all patients [3]. Although the majority of persons with hemophilia worldwide still lack access to prophylaxis, recent advancements, such as long-acting clotting factors, nonreplacement therapies, and gene therapy, have made the concept of “normalization” a realistic ambition in parts of the world with higher income. This concept was recently highlighted in several articles [4,5]. However, a number of significant challenges remain; there is no clear definition of normalization, no standardized way to evaluate it, no established strategies to achieve it, and potential risks are yet to be fully understood. It is time for healthcare professionals and the hemophilia community to reassess treatment goals and try to define what normalization truly means in hemophilia care.

## 2 Hemophilia: Definitions and Classifications

### 2.1 Impaired clotting factor production

Hemophilia is an X chromosome-linked congenital bleeding disorder characterized by a deficiency in clotting factor (F)VIII or FIX [2,6]. The conventional definition and classification of hemophilia are based on the severity of clotting factor deficiency measured in the blood [7]. Absolute or nearly complete absence of clotting factor (FVIII:C/FIX:C <1%) is termed severe hemophilia. This specific entity results in frequent spontaneous bleeding episodes, mainly affecting joints and muscles. Patients with moderate disease have levels between 1% and 5%, and patients with mild disease retain levels between 5% and 40%. In persons without hemophilia, FVIII concentration is between 50% and 150%, and it increases during multiple conditions, such as inflammation, infection, and pregnancy, as FVIII is one of the acute phase proteins [8]. Clotting factor activities can be easily measured using partial thromboplastin time-based one-stage clotting assays and chromogenic assays, in more specialized centers [9].

In hemophilia, there is a well-established correlation between blood clotting factor levels and bleeding phenotype, typically evaluated by the frequency of bleeding episodes and quantified by the annualized bleeding rate [10]. Generally, patients with severe hemophilia experience bleeding much more frequently than those with moderate hemophilia, supporting the rationale for converting severe cases to moderate by administering regular clotting factor infusions. However, such a factor-level-based approach should not be applied rigidly. Patients with moderate hemophilia (FVIII:C 1%-5%) and, rarely, those with mild disease can still experience frequent bleeding episodes or develop joint damage similar to that seen in severe cases [2,11,12]. Therefore, while clotting factor levels are crucial for classification, it is recommended to consider the patient’s bleeding phenotype and to manage individuals with a severe bleeding phenotype as if they had severe disease as assessed by clotting factor levels [11,13].

### 2.2 Defective thrombin generation

Hemophilia can also be understood as an impairment in thrombin generation, a characteristic it shares with various bleeding disorders, whether acquired or genetically inherited [14]. The cell-based model of coagulation divides hemostasis into 4 stages: initiation, amplification, propagation, and stabilization [15]. In recent years, there has been a significant advance in the understanding of coagulation with the introduction of a convergent model, which further extends coagulation beyond its boundaries by incorporating inflammation and immune activation, thereby addressing the shortcomings of earlier models exposed by the COVID-19 pandemic [16]. The primary objective of hemostasis is to generate enough thrombin. FVIII and FIX are crucial in the amplification phase of clotting, especially in tissues with low tissue factor levels, such as joints and muscles [17].

Contemplating hemophilia as a disorder of defective thrombin generation, rather than merely as a deficiency in FVIII or FIX, has traditionally been reserved for patients with inhibitors who require bypassing agents (eg, recombinant VIIa, activated prothrombin complex concentrate), which act by restoring thrombin generation. The focus on thrombin generation has grown recently with the development of nonreplacement therapies that either mimic FVIII or rebalance coagulation. These agents aim to enhance thrombin production in persons with hemophilia [18,19]. Thrombin generation assays have shown promise as useful tools for evaluating hemostatic function, but they are still not widely accessible, as there is currently insufficient data supporting routine clinical utility outside of the research setting. Therefore, further efforts are needed to standardize and validate these assays for routine clinical application [[20], [21], [22], [23]].

### 2.3 Impaired physical and mental health

Hemophilia has extensive implications that affect many facets of life [10,24,25]. Patients with hemophilia experience frequent bleeding episodes, particularly in joints and muscles, which can lead to long-term joint damage known as chronic arthropathy [26]. Such bleeds not only cause acute pain but also often lead to chronic pain, necessitating lifelong pain management strategies [27]. Additionally, severe bleeding episodes can be life-threatening and require immediate medical intervention. The physical impact of hemophilia often imposes limitations on daily activities and sports, as individuals avoid situations that might increase the risk of injury and subsequent bleeding [28,29]. These physical limitations extend into everyday life, affecting school attendance, work productivity, and even career choices [30]. Hemophilia also carries a significant financial burden due to the high costs of lifelong treatments, potential surgical interventions, and rehabilitation needs [31,32]. The psychosocial effects of hemophilia are equally profound [33,34]. Family dynamics may be affected, as loved ones play a crucial role in caregiving and emotional support [35]. Mental health challenges, including anxiety and depression, can also arise due to chronic pain, physical limitations, and concerns over the unpredictability of bleeding episodes. The cumulative impact of these factors on the QoL underscores the importance of holistic care for persons with hemophilia, addressing not only the physical symptoms but also the emotional and social challenges associated with the condition [36].

In summary, hemophilia encompasses much more than an increased bleeding tendency. It imposes significant physical challenges, including chronic pain, functional limitations, and potential disability, as well as substantial mental health impacts. The burden of treatment, coupled with lifestyle, educational, and occupational restrictions, profoundly affects not only patients but also their families and partners. New treatment options are progressively freeing persons with hemophilia from the physical and mental burden of both hemophilia and its demanding treatment [37].

## 3 Treatment Evolution and Rising Ambitions

### 3.1 Replacement therapy and normalization of clotting factor levels

Several therapeutics have been developed to provide persons with severe hemophilia with functional FVIII or FIX, which they lack almost completely [38]. Treatment options include whole blood, fresh frozen plasma, increasingly pure plasma-derived clotting factor concentrates, and finally, synthetic concentrates produced by harnessing biotechnology. In parallel, these treatments’ preventive (prophylactic) administration has emerged as the preferred therapeutic approach [39]. The treatment goal has been to convert severe hemophilia into a moderate form through intravenous injections administered several times per week, dictated by the short half-life of coagulation factor concentrates. Practically, the objective has been to ensure that the minimum concentration of FVIII or FIX measured before the next injection (ie, trough level) stays >1% [40].

Significant variation in the elimination rate of these factors justified the development of pharmacokinetic evaluation tools, allowing the adjustment of injection frequency and dosage based on individual pharmacokinetic parameters, thus enabling treatment personalization [41]. These strategies optimized the treatment and enabled achieving FVIII levels even >1%. Unsurprisingly, extensive research was dedicated to slowing the elimination of FVIII or FIX, leveraging multiple technologies (PEGylation, fusion with the Fc fragment of immunoglobulins, or albumin fusion) [42,43]. These technologies (PEGylation, fusion with the Fc fragment of immunoglobulins) facilitated a reduction in the frequency of intravenous FVIII injections from 3 times to 2 times per week in most persons with hemophilia. More recently, it became possible for some patients to maintain trough FVIII:C between 8% and 12% with extended half-life FVIII concentrates, administered according to individual pharmacokinetic characteristics [44].

For persons with severe hemophilia, the target trough level was traditionally set at FVIII:C >1% [45]. However, the time spent in this range was limited, especially with standard half-life FVIII concentrates. Thanks to therapeutic innovations, the target trough level has been increased. The most recently registered FVIII product, efanesoctocog alfa, was extensively modified by making it independent of endogenous von Willebrand factor by linking the FVIII-binding D'D3 domain of von Willebrand factor to B-domain deleted recombinant single chain FVIII Fc fusion protein and 2 XTEN polypeptides, starting a new era of high-sustained activity clotting FVIII concentrate [[46], [47], [48]]. With such therapy, an estimated therapeutic range of FVIII:C >40% for 4 of 7 days and FVIII:C >15% for 7 of 7 days can be achieved in 100% of adult patients, which represents an exceptional advancement [49]. For the first time, intravenous replacement therapy administered weekly enables correction of clotting factor concentration to a range considered normal for 4 days and in a range higher than that observed in the PROPEL study for >7 days. This is of particular interest for the most physically active persons with hemophilia who require better hemostatic protection. Moreover, treatment of bleeds or interventional procedures requiring higher factor levels can be managed with fewer infusions.

### 3.2 Nonreplacement therapies and normalization of coagulation

Motivated by the desire to avoid intravenous administration, maintain more constant hemostatic activity, and prevent neutralization by anti-FVIII and anti-FIX antibodies, several therapeutic options were developed [50]. These include a bispecific antibody that mimics the action of FVIII (emicizumab) and various hemostasis-rebalancing agents that enable increased thrombin formation in the absence of FVIII or FIX by targeting physiological inhibitors of coagulation (such as antithrombin, activated protein C, and tissue factor pathway inhibitor), including fitusiran, serpin-PC, concizumab, marstacimab, and others [19]. Recently, the very first medicines from this class, ie, marstacimab and concizumab, received approval from the United States Food and Drug Administration [51,52].

It is estimated that emicizumab provides coagulation equivalent to FVIII:C from 10% to 20% [[53], [54], [55], [56]]. The rebalancing agents partially restore thrombin generation, supposedly to an equivalent of FVIII:C ≥20% [9,57]. However, none of these molecules appear capable of fully normalizing the amount of thrombin generated. Second-generation bispecific FVIII-mimicking antibodies with enhanced affinity seem capable of providing higher thrombin generation [[58], [59], [60]]. Even when hemostasis is not fully normalized, less frequent and less invasive treatment regimens offer significant advantages, particularly by supporting a more normal daily routine and enabling persons with hemophilia to live with fewer burdens and fewer perceived limitations [61,62].

### 3.3 Gene therapy and normalization of clotting factor levels

One of the most innovative options in hemophilia treatment is gene therapy, which aims to induce endogenous production of FVIII or FIX by the liver [[63], [64], [65], [66]]. The current approach uses viral vectors with liver tropism that can carry modified *F8* or *F9* genes into hepatic cells, where they remain in episomal form. Eligibility criteria are strict, and not all patients respond to the treatment. The endogenous production of FVIII and, to a lesser extent, FIX is variable and appears to wane over time for FVIII. Based on the most recent data, 15% of patients treated with gene therapy for hemophilia A and 33% of those treated for hemophilia B maintain factor levels ≥40% [64,65]. Moreover, although some persons with hemophilia achieve sustained normalization of hemostasis after gene therapy, this approach cannot reverse irreversible complications and entails a significant long-term treatment burden due to the need for lifelong surveillance. On the other hand, published data demonstrate that even a partial improvement in FVIII:C levels—reaching the mild hemophilia range, and in some cases even the moderate range—is associated with fewer bleeding episodes and an improved QoL, with relatively few persons with hemophilia returning to regular prophylaxis [66]. To sum up, successful gene therapy currently represents the only strategy that can make a patient truly free of hemophilia, relieving them from both treatment and disease burden. In contrast, classical replacement therapies and nonreplacement strategies can sometimes render patients free from bleeding episodes but not free from hemophilia itself.

### 3.4 Do all treatment options carry the same potential for normalization of hemostasis?

**Figure 1:**
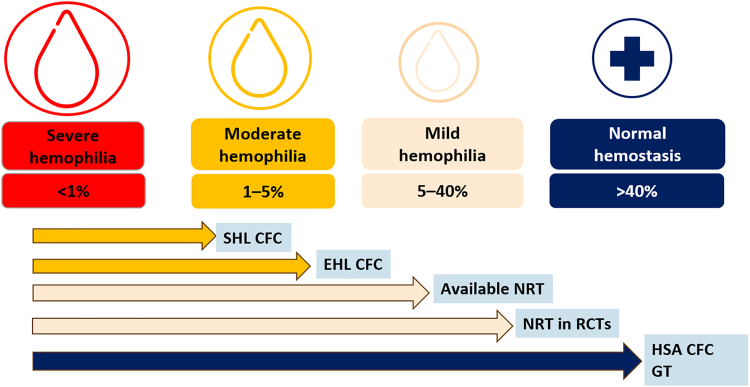
Visual and comparative representation of the measured or estimated hemostatic potential of currently available treatment options for hemophilia. Classical replacement therapy with standard half-life (SHL) and extended half-life (EHL) clotting factor concentrates (CFCs) allows the conversion of a severe hemophilia phenotype into a moderate one. Current nonreplacement therapies (NRTs) enable a shift from severe to mild hemophilia, with future NRTs expected to offer even higher potency. High-sustained activity (HSA) CFCs, considering factor III concentrations observed during the first 4 days postinfusion, and successful gene therapy achieved in ∼15% of treated patients with hemophilia A and 33% with hemophilia B based on current clinical trial data, can potentially allow full normalization of hemostasis. CFC, clotting factor concentrate; EHL, extended half-life; GT, gene therapy; HAS, high-sustained activity; NRT, nonreplacement therapy; RCT, randomized controlled trial; SHL, standard half-life.

All innovative hemophilia treatments share the goal of facilitating prophylaxis through either less frequent intravenous injections, subcutaneous injections, or even no injections at all for patients who benefit from effective gene therapy (Table; Figure 1). These treatments also commonly reduce or eliminate bleeding episodes by improving hemostasis through higher concentrations of clotting factors or enhanced thrombin generation. Given their high hemostatic efficacy, it is nearly impossible to compare new hemophilia treatments, as most patients experience <1 bleeding episode per year. In the absence of unrealistic randomized studies, it is currently impossible to establish the superiority of one treatment over another. The future should reveal whether these new treatments could differentially preserve joint health in patients with hemophilia by preventing microbleeds and the development of subclinical synovial lesions and synovitis, which may develop in patients treated with currently available prophylactic regimens [67,68].

**Table:** Hemophilia therapies and their potential to achieve normalization in different domains of hemophilia

Domains/Therapies	Clotting factor level	Thrombin generation	Bleeding phenotype	Physical and mental health^a^
SHL clotting factor concentrates	Full normalization, transient	Transient	Conversion into moderate phenotype in most patients	Limited improvement
EHL clotting factor concentrates	Full normalization for ∼20% of the time on treatment	Transient	Conversion into moderate phenotype in most patients	Moderate improvement
HSA FVIII concentrate	Full normalization for ∼50% of the time on treatment	50% of the time on treatment	Conversion into normal for ∼50% of the time; then mild phenotype	Great improvement
Approved bispecific antibodies	Limited to ∼15% of FVIII equivalent activity, sustained effect	Limited; sustained effect	Conversion into mild phenotype	Great improvement
Hemostasis-rebalancing agents	No impact on clotting factor concentration	Limited; sustained effect	Conversion into mild phenotype or even normal	Likely moderate-to-great improvement
Gene therapy (nonintegrating)	Fully normalized levels in the minority and a diminishing effect in the majority of persons with hemophilia	Fully normalized in the minority and a diminishing effect in the majority of persons with hemophilia	Severe into normal in the minority and a diminishing effect in the majority of persons with hemophilia	Excellent improvement in the minority and a diminishing effect in the majority of persons with hemophilia

FVIII, factor VIII; HSA, high-sustained activity; SHL, standard half-life.

a The entries in this column are based on personal opinions of authors and patients’ testimonials during consultations.

Collectively, the current data demonstrate that normalization of clotting factor concentrations can only be predictably and sustainably achieved in patients treated with high-sustained activity factor concentrate and unpredictably achieved in a limited number of eligible patients treated with gene therapy. The future should shed light on the ability of next-generation bispecific antibodies that mimic FVIII to induce thrombin generation in the normal range for all treated patients.

### 3.5 New therapies and normalization of life

Beyond hemostatic efficacy, all new treatments have a significant positive impact on patients’ QoL [69]. Multiple questionnaires are used in clinical studies and routine clinical practice to capture and assess this effect [24,25]. Patient testimonials during consultations probably provide the most convincing evidence of the new treatments’ capacity to positively transform patients’ lives in multiple dimensions [70]. New treatments cannot, however, eliminate the irreversible consequences of hemophilia, whether physical (arthropathy) or mental and psychological, such as the individual opportunities missed or made unattainable because of hemophilia.

Beyond normalizing factor concentration or thrombin generation, the new hemophilia treatments also hold enormous potential to normalize the lives of patients with hemophilia and their loved ones. However, this effect remains particularly challenging to objectively assess, and currently, it is not possible to compare the respective capacities of various new treatments to contribute to this mental liberation. Likewise, there is no systematic equivalence between the normalization of coagulation and the normalization of life. Indeed, for many patients with irreversible physical sequelae of hemophilia, the normalization of their coagulation will not equate to physical and mental normalization.

### 3.6 Controversy regarding the concept of normalization

The term “normalization” may be perceived as inappropriate or even offensive by some, as it implies a distinction between what is considered “normal” and “abnormal.” Therefore, it could be suggested to avoid the term “normalization” in the context of life, or physical and mental status, and exchange it with “improvement,” eg, in the QoL or physical or mental status. A possible explanation of the healthcare professionals’ temptation to use the term “normalization” in these contexts probably stems from long lasting linguistic traditions in the medical field of categorizing the results into normal, ie, within the reference range or “norm,” and abnormal or pathological. It is very important to understand that the final goal is to improve hemophilia care and the lives of persons with hemophilia. The achieved normalization of hemostasis and improved QoL does not negate the previous experiences of persons with hemophilia or their identity; therefore, it is crucial to highlight that there are absolutely no intentions to insult or humiliate anyone when reaching for improvement or ideally, normalization, equivalent to the status of unaffected individuals. Baas and colleagues [71] recently proposed that the interpretation of medical terminology should be context-specific, allowing for multiple uses to coexist, as long as each is appropriate to the function the concept (such as “cure”) serves in its particular setting. They also argued that more modest and clearly defined interpretations are necessary, especially in contexts such as health resource allocation and research funding decisions.

### 3.7 Normalization of coagulation and loss of protection against thrombosis

**Figure 2:**
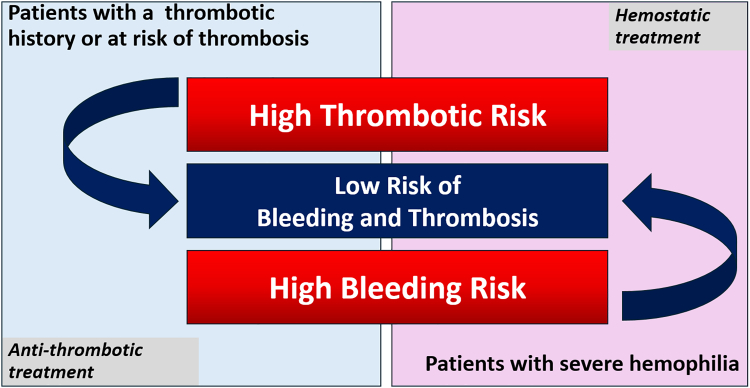
Hemostatic treatment in persons with hemophilia should aim to improve coagulation without increasing thrombosis risk, just as antithrombotic treatment for patients at risk of thrombosis should prioritize reducing thrombosis risk without increasing bleeding risk.

The hypocoagulability associated with hemophilia is clearly linked to a reduced risk of venous thrombosis [72,73]. Although patients with hemophilia are at risk for atheromatous disease due to the high prevalence of cardiovascular risk factors, the occurrence of atherothrombotic events or venous thrombosis remains rare, at least as long as patients remain in a hypocoagulable state [74]. However, such events have been observed with intensive factor concentrate treatment, the combination of emicizumab and activated prothrombin complex concentrate, some rebalancing agents, and in patients receiving gene therapy with supranormal levels of FVIII or FIX [64,65,[75], [76], [77]]. In the future, it may be advisable to avoid full normalization of factor levels or thrombin generation in patients at high risk of thrombotic complications or to balance normalization with concomitant antithrombotic therapy (Figure 2).

### 3.8 Normalization in women and girls with hemophilia

To the best of our knowledge, the concept of normalization in this population has not been explored. Most women and girls with hemophilia have mild FVIII or FIX deficiencies [78,79] and require normal factor levels to significantly improve their bleeding phenotype and QoL. When considering normalization of coagulation or thrombin generation, attention should be given to thrombotic challenges specific to women and girls with hemophilia, such as those related to contraception and pregnancy. It is also essential to identify and evaluate the specific impacts on women and girls to ensure safe and effective management strategies.

### 3.9 Normalization—why should we promote it?

Promoting normalization in hemophilia care offers significant benefits. For some patients, it represents a realistic and meaningful treatment goal, providing stable hemostatic control that can greatly enhance their QoL. Normalization also has the potential to address health equity in hemophilia, allowing more patients to achieve balanced and equitable health outcomes. Additionally, achieving normalization of hemostasis can have a profound impact on physical and mental well-being, liberating patients from the constant vigilance required to manage bleeding risks. Finally, normalized coagulation simplifies the management of hemostatic challenges, often eliminating the need for adjunctive therapies and reducing the overall complexity of treatment.

### 3.10 Normalization—current challenges and obstacles

Normalization in hemophilia treatment faces several challenges and obstacles. First, there is no standard definition of what normalization entails, complicating efforts to set clear targets. Additionally, normalization of hemostasis has limited or no impact on the irreversible consequences of hemophilia, which remains a concern for many patients. In some parts of the world, normalization is an unrealistic goal due to limited access to resources and advanced therapies. For nonreplacement therapies, evaluating normalization of hemostasis can be complex. It is also unachievable for patients with inhibitors and cannot be attained in all patients with all current treatment options. These challenges highlight the need for continued innovations and tailored approaches in hemophilia care.

## 4 Conclusions

Normalization appears to be the next ambition of hemophilia treatment. The full normalization of hemophilia would mean that patients could live their lives almost identically to people not suffering from bleeding disorders. Therefore, it is essential to prioritize the impact of treatments on both physical and mental well-being. Sustainable normalization of clotting factor levels is currently not achievable with most treatments and could be undesirable for some patients who encounter higher thrombotic risks. Achieving normal blood coagulation, defined as normal thrombin generation, is currently difficult to evaluate routinely. Normalization of life in terms of physical and mental well-being is becoming a reality for an increasing number of patients receiving innovative treatments. New assessment tools are urgently needed to better evaluate these improvements and capture the full impact on patients’ QoL.
